# Tracheal hemangioma: a case report and literature review

**DOI:** 10.1097/MS9.0000000000000850

**Published:** 2023-05-19

**Authors:** Hossein Sadidi, Pegah Bahrami Taqanaki, Hamed Amirfakhrian, Reza Rezaei

**Affiliations:** a Thoracic Surgery Department of Ghaem Hospital, Mashhad University of Medical Sciences; b Mashhad University of Medical Sciences; c Endoscopic and Minimally Invasive Surgery Research Center, Mashhad University of Medical Sciences, Mashhad, Iran

**Keywords:** case report, hemangioma, stridor, tracheal hemangioma

## Abstract

Hemangiomas are the most common vascular tumors in children. Although hemangiomas are common, they are rarely seen in areas like the trachea and larynx.The most common clinical manifestations in tracheal hemangiomas are stridor and respiratory distress. The main diagnostic method is bronchoscopy. Other imaging techniques like computed tomography scans and MRIs are also helpful. Various treatment options are now used for treating the disease, including beta blockers like propranolol, local and systemic steroids, and surgical resection.

**Case presentation:**

An 8-year-old boy with the chief complaint of severe progressive dyspnea and a history of neonatal postbreastfeeding cyanosis was admitted. On physical examination, he had tachypnea, and stridor was heard upon auscultation. There was no history of fever, chest pain, or coughing. He underwent a rigid bronchoscopy followed by a neck computed tomography scan. The results indicated a soft tissue mass with a vascular nature. An MRI of the neck confirmed the diagnosis of tracheal hemangioma. The mass was not resectable during surgery; hence, angioembolization was carried out. Treatment was successful and there was no recurrence on the follow-up.

**Clinical discussion:**

Based on the findings in this literature review tracheal hemangiomas present with stridor, progressive respiratory distress, dyspnea, hemoptysis, and chronic coughs. Advanced tracheal hemangiomas commonly do not reduce in size by themselves and need treatment. A close follow-up ranging from 3 months to 1 year is recommended.

**Conclusion:**

Although tracheal hemangiomas are rare they should be considered in the differential diagnosis of severe dyspnea and stridor.

## Introduction

Highlights

- Hemangiomas are the most common benign soft tissue tumors in children. Even though their most common sites are the head and neck, they are rarely seen in the trachea and larynx.
- Tracheal or laryngeal hemangiomas can significantly affect patients’ lives, and even cause respiratory distress and arrest. Hence, proper diagnosis and treatment of these diseases are crucial. Tracheal hemangioma’s most common signs and symptoms are stridor, respiratory distress, progressive dyspnea, chronic cough, and hemoptysis.
- The main diagnostic tool for this disease is bronchoscopy.
- Treatment options vary from beta blockers like propranolol, local and systemic steroids, to surgical resection.

Vascular diseases in children and infants include vascular tumors and malformations. Hemangiomas are the most common among vascular tumors and also the most common benign soft tissue tumor in infants^1,2^. Infantile hemangiomas occur in nearly 4–5% of infants, which may be present at birth or shown later in life^3^. Hemangiomas go through an involution stage once they have reached their complete growth near one year of age and it may take from months to years; however, a small percentage of hemangiomas never go through this stage and do not become smaller in size^3,4^. The most common areas of involvement are the head and neck with the subglottic region being the most involved^4^. Although hemangiomas usually do not require special intervention, factors such as hemangioma size, growth rate, and location can change this^5^.

Hemangiomas residing in the larynx or trachea, although rare, need close monitoring and intervention as they may lead to irreparable and life-threatening consequences. Tracheal hemangiomas present in various ways in children and adults. Dyspnea, stridor, chronic cough, and obstructive respiratory disease are the most common signs and symptoms of tracheal hemangiomas in children. The main diagnostic tool for tracheal hemangiomas is bronchoscopy^6^. Several different approaches have been suggested over time for the treatment of tracheal hemangiomas including steroid injections, endoscopic and open excisions, and medications like systemic steroids and vincristine^5^.

Here, we present a case of an 8-year-old with intraluminal tracheal hemangioma presenting with respiratory distress to an academic tertiary center, which was managed with three sessions of angioembolization.

This manuscript was prepared following the Surgical CAse REport (SCARE) guidelines^7^.

## Case presentation

An 8-year-old boy without any documented medical or surgical history was admitted to a tertiary academic center with the chief complaint of progressive dyspnea. His parents mentioned that he used to suffer from postbreastfeeding cyanosis in infancy and also after crying. In the years after infancy and before school age the patient’s symptoms were described as exertional dyspnea becoming worse by exercising and walking accompanied by fast heartbeats and increased shallow breathes. As the patient had reached the school age, his dyspnea had become more severe with stridor heard while breathing. His dyspnea and stridor had progressed in the last 3 months. He had not experienced fever, chest pain, weight loss, and coughing in the course of his illness.

In general appearance, he was a child with a height of 125 cm and a weight of 27 kg, and physical examination revealed tachycardia (heart rate: 120), tachypnea (respiratory rate: 30), mild hypotension ( blood pressure: 90/70), and low oxygen saturation (Spo_2_: 85%). Laboratory results were normal and no anemia or leukocytosis was seen.

**Figure 1:**
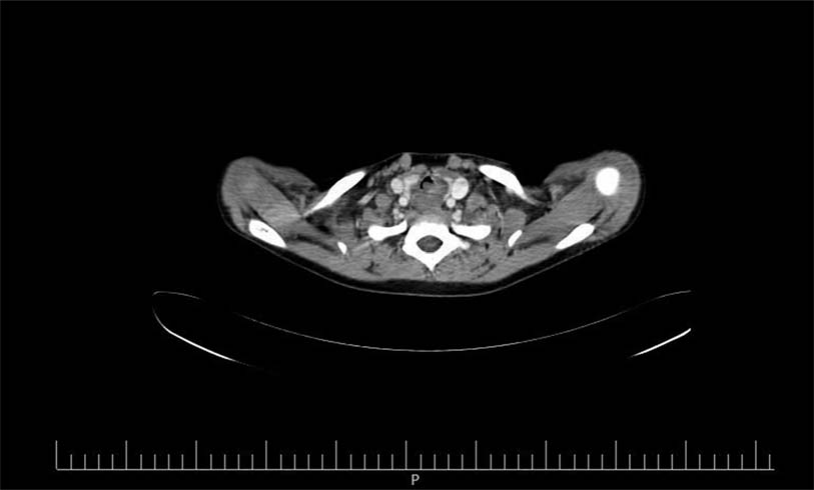
Demonstrate the neck CT scan of the patient demonstrating a vascular mass behind the larynx and cervical trachea.

**Figure 2:**
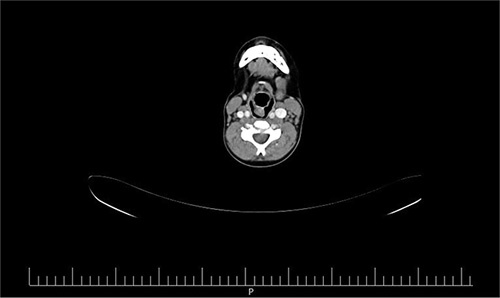
Demonstrate the neck CT scan of the patient demonstrating a vascular mass behind the larynx and cervical trachea.

**Figure 3:**
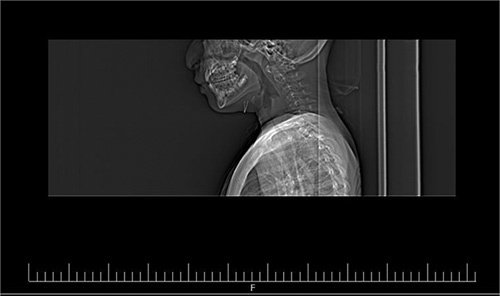
Demonstrate the neck CT scan of the patient demonstrating a vascular mass behind the larynx and cervical trachea.

The patient underwent a rigid bronchoscopy. Bronchoscopy findings included normal tracheal mucosa and a soft tissue mass that could be compressed with the bronchoscope. Afterward, a neck computed tomography (CT) scan was performed revealing a vascular mass behind the larynx and cervical trachea (Figs. 1–3). Subsequently, surgery is performed for investigating the mass in the patient’s neck. It was reported during the operation that the mass is of a vascular nature but resection could not be done. A tracheostomy was performed by the surgeon in the same session.

**Figure 4:**
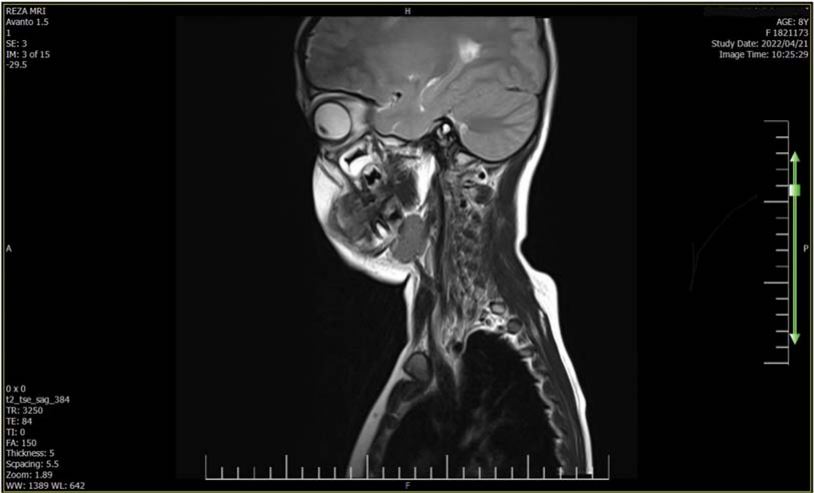
Present the MRI images which reveal a multiloculated nodular lesion in the intraluminal part of the trachea and also partly in the infra-glottic area, measuring 12×8 mm and taking up half of the tracheal lumen.

**Figure 5:**
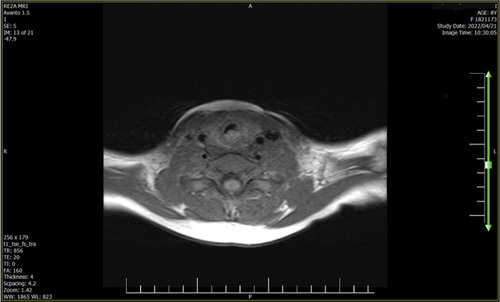
Present the MRI images which reveal a multiloculated nodular lesion in the intraluminal part of the trachea and also partly in the infra-glottic area, measuring 12×8 mm and taking up half of the tracheal lumen.

He was transferred to the radiology department for an MRI with contrast. At MRI, a multiloculated nodular lesion in the intraluminal part of the trachea and also partly in the infra-glottic area, measuring 12×8 mm and taking up half of the tracheal lumen, was found. The lesion was hypo-signal in the T1 sequence, hyper-signal in the T2 sequence, and heterogeneous after the contrast injection (Figs. 4 and 5). MRI findings confirmed the diagnosis of hemangioma.

Three sessions of angioembolization were carried out for the patient, and the patient’s progress was controlled with MRI. Finally, after hemangioma atrophy tracheostomy was extracted.

He was given guaifenesin every 8 h and discharged from the hospital. At 3 month follow-up, the patient’s symptoms were significantly improved and repeated bronchoscopy did not demonstrate any recurrence.

## Discussion

Hemangioma is a soft tissue tumor caused by hyperplasia of the endothelial cells. Hemangiomas are either congenital or infantile. Infantile hemangiomas appear in the first 8 weeks of birth and grow rapidly until 6–12 months of age, and then they usually shrink in a natural and physiological way and disappear completely. Congenital hemangiomas; however, are fully developed lesions at the time of birth^8^. Hemangiomas are classified by the extent of tissue involvement as focal, segmental, and indeterminate, and by the depth of the tissue affected as deep, superficial, and mixed^8^.

Tumors of the tracheobronchial tree are a rare concept in children and are divided in two groups of benign and malignant lesions. The most common benign tumors of the tracheobronchial tree in children are hemangiomas and squamous papilloma^9^. Various factors may lead to the formation of these hemangiomas consisting of hormonal imbalance in pregnancy, infections, arteriovenous malformations, and vascular growth factors alteration^10^.

The low prevalence of this disease causes a delay in its diagnosis, as seen in this case. The presence of a mass in the trachea can cause symptoms such as stridor, cough, shortness of breath exacerbated by exercise, and hemoptysis (in the case of hemangiomas)^11^. A tracheal hemangioma can present as intractable asthma and may lead to respiratory failure and even death in severe cases.

As the signs and symptoms of a tracheal hemangioma are not specific to the disease the initial diagnostic tool used for finding the etiology is a chest radiograph as in all the respiratory diseases. CXR is commonly not diagnostic for the disease but may exclude other differential diagnoses. Further diagnostic tools that have been proven to have appropriate sensitivity and specificity in diagnosing the lesion are the CT scan, MRI, and bronchoscopy^9^. Rigid bronchoscopy and flexible endoscopy are used to retrieve samples and histopathologic assessments confirm the diagnosis; however, some studies start the treatment with imaging findings and do not use pathologic samples for confirmation^9^.

The case presented in the current study is a display of a tracheal mass presenting itself with respiratory distress and severe dyspnea.

We used three databases of PubMed, Scopus, and Web of Science to conduct a literature review. Keywords used were ‘Tracheal Hemangioma’ and ‘Hemangioma of Trachea’ and all studies from 1 January 2013 to 5 March 2023 were included initially. The initial number of articles included was 368, which was then reduced to 216 after deleting the duplicates. After reviewing the remaining articles, irrelevant articles (articles discussing nontracheal hemangiomas, nonhemangioma tumors mimicking hemangiomas, subglottic hemangiomas not radiating to the trachea, cancer treatments, and articles not reporting cases of the issue) were excluded. The remaining articles were assessed for eligibility to be included in this study and 20 articles were excluded due to the age of the patients, as only pediatric case series or case repots are included in the current study. Although one study was conducted on patients with subglottic hemangiomas with reported tracheal stenosis, with the aim of investigating their perioperative airway managements, it did not specify the exact location of the hemangioma and the number of patients with tracheal hemangioma was not reported, hence the study was excluded from this review^12^. Finally, 15 studies were included in the review including 12 case reports and 3 case series. The summary of the studies are displayed in Table 1.

**Table 1:** The summary of the studies included in the review

References	Study type	Age/Sex	Past medical History	Chief complaint	Physical Examination	Imaging findings and histopathology assessments	Diagnosis	Size	Treatment	Adverse events	Follow-up	Recurrence
D. Ho-Wo-Cheong, et al (Canada)^13^	Retrospective case series on patients diagnosed with Synchronous Airway Lesions(SALs).	One patient was diagnosed with tracheal hemangioma among 73 patients diagnosed with SALs (The age and sex of the patient was not specified).	Not mentioned	Not mentioned	Not mentioned	Not mentioned	Tracheal hemangioma	Not mentioned	Not specified	Not mentioned	Not mentioned	Not mentioned
D. Jacobson, et al (Canada)^14^	Case report	First weeks of life but the exact age is not mentioned/ Male.	Premature delivery(28 weeks), Respiratory distress syndrome, Hyperbilirubinemia, Probable laryngomalacia.	A red oval shaped plaque with sharp borders and rough surface on neck which had increased in size.	Biphasic stridor	Flexible nasopharyngolaryngoscopy ruled out supraglottic origin Bronchoscopy revealed a hemangioma obstructing 15% of the trachea with the lesion being on the posterior tracheal wall.	Tracheal hemangioma	4×5 cm	Propranolol (2 mg/kg/day) was started on the patient’s 34th day of life with an increase in dose every 2 days.	Bradycardia on day 26 of treatment which led to stoppage of treatment for several days.	Decrease in size of the tumor (2×1 cm) after 8 weeks of treatment. The patient was visited at 40 weeks corrected gestational age and had improved in signs and symptoms.	None
A. Kilicaslan, et al (Turkey)^15^	Case report	42 months old/ Female	PHACE syndrome (posterior fossa anomalies, hemangioma, arterial anomalies, cardiac anomalies, and eye anomalies).	Stridor	Stridor	Right internal carotid artery agenesis and hemangiomas in the left parotid gland and laryngo-tracheal space.	PHACE syndrome with multiple hemangiomas on her face and neck and the laryngo-tracheal area.	Not mentioned	Propranolol with systemic steroids for 24 h then surgical excision was performed.	None	Uneventful after the surgery.	Not mentioned.
R. G. Elluru, et al (USA)^16^	Case series	One patient was diagnosed with tracheal hemangioma among 27 patients diagnosed with SALs.	Not specified	Not mentioned	Stridor	Localized tracheal infantile hemangioma.	Tracheal hemangioma.	Not mentioned	Propranolol alone	Not mentioned	The patient was followed by bedside endoscopy, however it was not specified in the study whether the patient had responded well to the treatment.	Not mentioned
J. Choi, et al (South Korea)^17^	Case report	2 months/ Female	Respiratory distress 20 days after birth. Cutaneous hemangiomas on the right cheek, perioral, and mandibular areas Bronchiolitis.	Dyspnea and stridor	Tachypnea, subcostal retractions, coarse breath sound, and inspiration stridor.	A swollen vocal cord and narrowed subglottic space with superficial erosions in bronchoscopy, 3D-CT/bronchoscopy showed a tracheal hemangioma with 2.73 mm thickness and 4.08 mm airway narrowing.	Tracheal hemangioma	2.73 mm thickness	Oral prednisolone 10 mg/day was started initially. Due to great response to the initial dosage of prednisolone patient was discharged with lower dose of prednisolone (7.5 mg/kg/day). On day 17 the dose was further reduced to 5 mg/kg/day which led to respiratory distress of the patient therefor continuing the treatment with the initial 10 mg/kg/day dose/ Nebulized budesonide/ Oral propranolol.	None	Patient follow-up was measured in three ways: Response to prednisolone 3D-CT/bronchoscopy which showed reduced tumor thickness and tracheal narrowing Bronchoscopy which revealed improvement in the narrowing and reduced resistance while entering the trachea.	Not mentioned
C. Robitaille, et al (Canada)^18^	Case report	15 years old/ Female	None	Acute dyspnea and hemoptysis.	No significant finding	CT scan showed 11×1 mm polypoid lesion arising from the right anterolateral wall of the upper trachea with focal contrast enhancement, Initial Bronchoscopy revealed a red pedunculated lesion with bright blood on its surface, located 20 mm under the vocal cords protruding into the tracheal lumen, with an 80% obstruction of the trachea A hemorrhagic area under the fibrous capsule of the lesion was observed.	Capillary hemangioma of the trachea.	11×11 mm	Surgical excision	None	The patient had improvement in her symptoms immediately after the operation.	None
J. C. Oliveira, et al (Portugal)^19^	Case report	12 months/ Male	Respiratory distress	Dyspnea and stridor	Biphasic stridor increasing in supine position/ Posterior cervical hemangioma.	Flexible bronchoscopy showed a multilobulated subglottic hemangioma obstructing more than 70% of the tracheal lumen. MRI revealed an angiomatous malformation.	Multilobulated subglottic hemangioma protruding the trachea.	Not mentioned	Systemic and local steroids Laser ablation at 5 years old.	None	The patient was followed for 14 years and was symptom free at that age.	None
M. A. Özgül, et al (Turkey)^20^	Case report	12 years old/ Male	None	Recurrent hemoptysis since 2 years ago.	No significant findings	CT scan of thorax revealed a polypoid lesion on the left lateral wall of the proximal trachea Rigid bronchoscopy demonstrated a polypoid lesion with pedicle on the proximal trachea.	Tracheal capillary hemangioma	Not mentioned	Interventional bronchoscopy ( excision with electrocautery).	None	The patient was followed 6 months later and was doing well.	None
H. Ajmi, et al (Tunisia)^21^	Case report	2 months/ Female	None	Respiratory distress Stridor.	Tachypnea Stridor more prominent on inspiratory phase bronchial wheezing.	Direct laryngoscopy and MRI were carried out and both revealed a soft, subocclusive lesion obstructing almost all the laryngo-tracheal airway.	Subglottic hemangioma protruding and obstructing the trachea.	Not mentioned	The patient was initially treated with nebulized epinephrine and steroids With the aim of treating the respiratory distress and after the definite diagnosis she was started on propranolol 1 mg/kg/day (increased to2 mg/kg/day later).	None	The patient was fully recovered 1 year later; however, the treatment was continued for 2 years.	None
L. A. Petrauskas, et al (USA)^22^	Case report	6 weeks/ Not mentioned	PHACE syndrome	Stridor	Severe biphasic stridor Retro-sternal retractions and respiratory distress Right sided facial hemangioma that extended to the right chest and arm.	Rigid bronchoscopy revealed a circumferential subglottic hemangioma, along the posterior tracheal wall to the carina.	Circumferential subglottic hemangioma expanding to trachea.	Not mentioned	Submucosal resection with laryngo-tracheal reconstruction.	None	The patient was followed for 8 months and was doing well until then.	None
D. Y. Yang, et al (China) ^23^	Case report	9 months/ Male	None	Dyspnea	Wheezing and severe respiratory distress.	High resolution computed tomography (HRCT) revealed a round lesion in the middle segment of trachea With filling defect of trachea Abdominal CT also showed the same lesion in the liver Bronchoscopy revealed a mass full of vessels in the middle of the trachea, originating from the right wall , which blocked almost all the airway.	Tracheal hemangioma	6.5 mm×7.1 mm×8.3 mm	Propranolol 2 mg/kg/day three times a day for 17 days Dexamethasone (0.5 mg/ kg/d, q.d) for 4 days Bronchoscopic excision with cryotherapy.	After several days of treatment with propranolol and seemingly reduction in the hemangioma, the patient developed dyspnea and fever.	On follow-up CT scan was done day 17 of the treatment which showed reduction size of the lesion, however later it was discovered that the lesion had increased in size again After surgical excision of the hemangioma bronchoscopy was done 1 weeks and 3 months later showing complete improvement.	None
Y. Fujita, et al (Japan) ^24^	Case report	1 month/ Female	None	Severe dyspnea	Stridor and severe respiratory distress.	Bronchofiberscopy revealed bulging in the subglottic tracheal membrane Primary neck MRI showed a hyper-signal (T2) compressed mass Neck MRI 13 days after intubation did not show any lesions.	Hemangioma or Lymphangioma Exact diagnosis was not decided.	Not mentioned	Intubation No specific treatment was done for the subglottic mass and the lesion was reduced in size naturally.	None	Patient was followed for 5 months and did not show any symptoms.	None
J. A. Czechowicz, et al (USA) ^25^	Retrospective case series on children with PHACE syndrome.	10% of the 55 patients were diagnosed with tracheal hemangioma (the age and sex of the patients was not specified).	PHACE syndrome	Not mentioned	Not mentioned	Bronchoscopy detected the location of the mass in the trachea in 10% of the cases.	Tracheal hemangioma		Propranolol 2 mg/kg/day. Other treatment options including flexible bronchoscopy and surgical laryngoscopy or bronchoscopy were done for number of patients; however, it was not specified whether they were done for the tracheal hemangioma patients.	None	Not mentioned	Not mentioned
R. Y. Wang, et al(USA)^26^	Case report	10 years old/ Female	Adenotonsillectomy	Chronic barking cough Vomiting Recurrent pneumonia since infancy.	No significant findings.	Tracheoscopy showed a subtle sessile lesion of the posterior tracheal wall Histopathalogic assessment of the lesion revealed lobular aggregates of capillary-sized vessels with endothelial and inflammatory cells CT scan of the neck with contrast demonstrated a hyperdense mass in the posterior trachea at the level of C7.	Tracheal Lobular Capillary Hemangioma	4×2×10 mm	Rigid tracheoscopy and laser ablation	None	The patient was followed 4 months later with physical examination and repeated tracheoscopy which showed improvement in signs and symptoms and no mass.	None
W. Barbaria, et al ^27^ (Tunisi)	Case report	8 years old/ Female	None	Coughing blood	No significant findings	CT scan of the chest showed a polypoid lesion on the right anterolateral wall of the proximal trachea Bronchoscopy confirmed the CT scan findings The second bronchoscopy after 2 months of treatment with propranolol indicated the failure of the beta blocker treatment in reducing the size of the mass Histopathologic assessments confirmed the diagnosis of hemangioma(9 mm vascular proliferation).	Tracheal hemangioma	9 mm	Treatment with beta blockers was initially started which did not succeed The tumor was finally removed by electrical excision.	None	The patient was followed 24 months later with complete improvement and no hemoptysis Control bronchoscopy was not done.	None

To summarize, a total of 19 children were reported to have tracheal hemangiomas or other subglottal lesions protruding to the trachea. The country distribution of the cases were as follows: USA 42.1% (8/19), Canada 15.7% (3/19), Turkey 10.5% (2/19), Tunisia 10.5% (2/19), Japan 5.2% (1/19), China 5.2% (1/19), and Korea 5.2% (1/19) . Four studies (eight patients) did not mention the age of the patients and the average age in other studies was 55.4 months (from 6 weeks to 15 years) with 54.54% of the cases being 1 year old or younger. There was a slight prevalence of the disease in female with the male to female ration being 4:7. The reported comorbidities and past medical histories included prematurity 5.2% (1/19), hyperbilirubinemia 5.2% (1/19), laryngomalacia 5.2% (1/19), respiratory distress early in life 15.7% (3/19), PHACE syndrome 36.8% (7/19), and a history of adenotonsillectomy 5.2% (1/19). It should be noted that one case suffered from severe prematurity and respiratory distress syndrome at birth, hyperbilirubinemia, and laryngomalacia at the same time. Two studies did not mention the past medical history of the patients and six patients did not have any comorbidities or prior medical conditions.

Among 13 patients whose signs and symptoms were recorded, the most common signs and symptoms were stridor 69.23% ( 9/13), respiratory distress 38.46% (5/13), and hemoptysis 23.07% (3/13). Other complaints consisted of dyspnea, chronic coughing, recurrent respiratory infections, and sporadic fascial and cervical hemangiomas. The diagnostic tools used in all studies were mainly a CT scan, an MRI, bronchoscopy, and histopathologic assessments for confirmation. One study confirmed the accuracy of the 3d-CT/bronchoscopy diagnosis for the tumor.

Only one case series did not specify the treatment option used for the patient with tracheal hemangioma. The main treatment option observed in the studies was the use of Propranolol 61.11 (11/18), which in 10 patients led to improvement of the signs, symptoms, and the size of the tumor. There was a failure of the propranolol regimen in one study, which led to electrical excision of the lesion. Laser ablation, steroids therapy, and surgical removal of the lesion were reported in 27.77% ( 5/18), 16.66 (3/18), and 16.66 (3/18), respectively. Natural shrinkage of the tumor was reported in one case.

There was no report of a tracheal hemangioma from Iran in the past 10 years, therefore making this case report the only reported tracheal hemangioma in the past 10 years in Iran.

The study searching was conducted by two separate individuals; hence, improving the accuracy of the search results. All included studies were obtained in full-text form and were in English language; hence, no exclusion was needed in this part.

The limitations of this literature review include a publication bias as all studies are case reports and series. The results of this study depend on the quality of the literature search.

## Conclusion

Tracheal hemangiomas are a rare concept, especially in children. These tumors present with stridor, progressive respiratory distress, dyspnea, hemoptysis, and chronic coughs. Propranolol has been reported to have great efficacy in improving the symptoms and reducing the size of the lesion. Systemic and localized steroids, surgical removal, and laser ablation are other successful methods for treatment. It is not common for a tracheal hemangioma presenting with severe symptoms to shrink naturally. Although there is not sufficient literature, based on this literature review, recurrence is not a common finding after the mentioned treatments as none of the cases reported showed recurrence. A close follow-up ranging from 3 months to 1 year is recommended.

## Ethical approval

NA.

## Consent

No personal information of patients have been revealed in the study.

## Sources of funding

None.

## Funding

None.

## Author contribution

R.R., H.S.: study concept or design; H.S., P.B.T., H.A.: data collection; R.R.: supervision; H.S., P.B.T., H.A.: data analysis or interpretation; P.B.T.R., R.R., H.A.: writing the paper.

## Conflicts of interest

None.

## Research registration unique identifying number (UIN)

None.

## Guarantor

Reza Rezaei.

## Data availability statement

All the required data are available in the manuscript.

## Provenance and peer review

Not commissioned, externally peer reviewed. Tracheal hemangioma: a case report and literature review.

## Assistance with the study

None.
